# High‐fat diet effects on amniotic fluid volume and amnion aquaporin expression in non‐human primates

**DOI:** 10.14814/phy2.13792

**Published:** 2018-07-23

**Authors:** Cecilia Y. Cheung, Victoria H. J. Roberts, Antonio E. Frias, Robert A. Brace

**Affiliations:** ^1^ Division of Maternal‐Fetal Medicine Department of Obstetrics and Gynecology Oregon Health and Science University Portland Oregon; ^2^ Division of Reproductive and Developmental Sciences Oregon National Primate Research Center Portland Oregon

**Keywords:** Amniotic fluid volume, aquaporins, high‐fat diet, monkey

## Abstract

Western style, high‐fat diet (HFD) and associated high lipid levels have deleterious effects on fetal and placental development independent of maternal obesity and/or diabetes. Our objectives were to determine whether HFD without development of obesity would alter amniotic fluid volume (AFV) and amnion aquaporin (AQP) expression in a non‐human primate model. Japanese macaques were fed either a control diet or HFD before and during pregnancy. The four quadrant amniotic fluid index (AFI) was used as an ultrasonic estimate of AFV at 120 days gestation. Amnion samples were collected at 130 days gestation by cesarean section and AQP mRNA levels were determined by quantitative RT‐PCR. Similar to that in human, AQP1, AQP3, AQP8, AQP9, and AQP11 were expressed in the macaque amnion with significant differences in levels among AQPs. In macaque, neither individual AQPs nor expression profiles of the five AQPs differed between control and non‐obese HFD animals. There were regional differences in AQP expression in that, AQP1 mRNA levels were highest and AQP8 lowest in reflected amnion while AQP3, AQP9, and AQP11 were not different among amnion regions. When subdivided into control and HFD groups, AQP1 mRNA levels remain highest in the reflected amnion of both groups. The HFD did not significantly affect the AFI, but AFI was positively correlated with AQP11 mRNA levels independent of diet. Collectively, these data suggest that HFD in pregnant non‐obese individuals may have at most modest effects on AFV as the AFI and amnion AQP expression are not substantially altered.

## Introduction

Consumption of a western‐style, high‐fat diet (HFD) during pregnancy has been shown to alter maternal and fetal metabolism (Frias and Grove 2012). The associated high lipid levels cause deleterious effects on fetal and placental development leading to long‐term health consequences (O'Tierney‐Ginn et al. 2015; Roberts et al. 2015; Harris et al. 2016; Lowensohn et al. 2016; Contu and Hawkes 2017). These effects often occur independent of the development of obesity. Although unexplored, one potential contributor to the adverse effects of HFD on the fetus and placenta could be an abnormal amniotic fluid volume (AFV). It has been well‐documented that alterations in AFV during human pregnancy adversely affect maternal and fetal outcome. Both oligohydramnios and polyhydramnios are associated with pregnancy complications including preterm birth, low birth weight, abnormal fetal lie, placental abruption, postpartum hemorrhage, and cesarean delivery (Locatelli et al. 2004; Hamza et al. 2013; Morris et al. 2014). However, this issue can be complex because pregnant subjects who are on HFD and consequent obesity often have comorbid gestational diabetes and that, in turn, is associated with elevated AFVs compared to normal (McMahon et al. 1998; Abele et al. 2012).

Although an understanding of the regulation of AFV in humans is necessary for mitigating the adverse effects of aberrant AFVs on the fetus and newborn, much of our understanding of the mechanisms that regulate AFV have been derived from studies in fetal sheep (Brace and Cheung 2014; Brace et al. 2014). In the ovine experimental model, AFV is primarily determined by the rate of fluid transfer across the amnion (Brace and Cheung 2014; Brace et al. 2014). However, the regulators of this trans‐amnion transport pathway have not been clearly established. Aquaporins (AQPs) are transmembrane proteins that facilitate transport of water and small solutes across semi‐permeable membranes. The presence of AQPs in the amnion indicates that they most likely participate in trans‐amnion amniotic fluid transport (Liu and Wintour 2005; Mann et al. 2005; Beall et al. 2007). Consistent with this concept is our recent finding that AQP1 expression in the ovine amnion was positively correlated with the rate of fluid transfer between the amniotic fluid compartment and fetal intramembranous vasculature, thus potentially participate in AFV regulation (Cheung et al. 2016).

In humans, five aquaporins (AQP1, AQP3, AQP8, AQP9, and AQP11) have been identified in the amnion (Prat et al. 2012). The amnion expression profiles reveal differences in expression level of individual AQPs as well as among amnion regions (Bednar et al. 2015). Functionally, AQP1 is a highly selective water transport channel whereas AQP3 and AQP9 are aquaglyceroporins that facilitate glycerol transport in addition to water. As such, the AQPs expressed in the amnion may play an important role not only in water movement but also in transfer of other solutes such as glycerol. Past studies on the role of AQP in AFV regulation were mostly conducted in pregnant sheep, however, several differences between the ovine model and human pregnancies present challenges for translational studies. Non‐human primates would conceptually provide a more appropriate model for investigating the regulation of AFV in humans; yet there have been very few relevant studies in fetal monkeys. One study reported that intramembranous fluid exchange occurs rapidly between amniotic fluid and fetal blood across the fetal surface of the rhesus placenta (Gilbert et al. 1997). This is similar to the rapid uptake of amniotic fluid into the fetal circulation shown in sheep fetuses (Brace et al. 2014). The specific role of AQPs in this rapid fluid transfer has yet to be established but may be important as AFV is elevated in AQP1 knockout mice (Mann et al. 2005).

Our group has developed a well‐established Japanese macaque HFD model which has been extensively utilized for pregnancy and offspring outcome studies (McCurdy et al. 2009, 2016; Grayson et al. 2010; Nicol et al. 2013; Pound et al. 2014). In this model, animals chronically fed a HFD typically segregate into two phenotypes: HFD‐Sensitive (HFD‐S) animals that become obese and have dysregulation of insulin sensitivity analogous to diabetes in humans, and HFD‐Resistant (HFD‐R) animals that remain lean and have normal insulin function (McCurdy et al. 2009; Frias et al. 2011). We have previously demonstrated increased placental inflammation and infarction in this model with effects that are observed due to diet alone and independent of maternal obesity (Frias et al. 2011). Since approximately half of the macaques on the HFD prior to and during pregnancy do not become obese and do not have dysregulation of insulin function (HFD‐R), this phenotype may possess an endogenous protective mechanism that serves to reduce the impact of increased fat intake, thus providing a unique opportunity for determining the effects of HFD alone on AFV and amnion AQPs in non‐obese and non‐diabetic subjects. The monkey placenta is of particular interest because of the presence of a bi‐discoid placenta with the secondary placenta generally smaller in diameter than the primary (Grigsby 2016), thus raising the possibility of regional differences in AQP expression in relation to each placental disc.

In this study, in order to explore the effects of a HFD on amniotic fluid status and to advance knowledge in the regulation of AFV, we sought to determine whether a HFD would alter AFV and amnion AQP expressions in macaques that were non‐obese and resistant to the HFD. We hypothesized that the five AQPs expressed in human amnion are also expressed in macaque amnion and that the profile of expression would be similar. In addition, we postulated that regional differences in AQP mRNA levels would exist in macaque amnion. Furthermore, we hypothesized that a HFD would reduce AFV in HFD‐R animals in parallel with an increased expression of AQPs within the amnion.

## Materials and Methods

### Animal preparation

All animal protocols were approved by the Institutional Animal Care and Utilization Committee (IACUC) of the Oregon National Primate Research Center (ONPRC), and guidelines for humane animal care were followed. The ONPRC abides by the Animal Welfare Act and Regulations enforced by the USDA.

Pregnant Japanese macaques (*Macaca fuscata*) underwent an ultrasound examination at approximately 120 days gestation (term = 175 days). In brief, after overnight food withdrawal, sedation was induced with a 5‐ to 15‐mg/kg intra‐muscular injection of ketamine. Subjects were positioned in dorsal recumbence, and physiological vital signs were monitored throughout the procedure. The four quadrant amniotic fluid index (AFI) was measured (GE Voluson 730 Expert; Kretztechnik, Zipf, Austria) by one ultrasonographer (AEF) and used as an index of AFV.

As described in detail elsewhere, the animals were maintained on either a control, standard monkey chow diet (*n* = 8, 14% fat) or chronically on a HFD (*n* = 10, 36% fat. Purina Mills, Inc., St. Louis, MO) prior to and during pregnancy (Harris et al. 2016). The composition of this diet constituted a typical western style diet with respect to the saturated fat content, and was matched in micronutrient content to the standard control chow diet. Both diets were sufficient in vitamin, mineral, and protein content for normal growth. Only HFD animals resistant to the diet were included in this study. At necropsy, amnion tissues were obtained from the primary placental, secondary placental, and reflected amnion. Because this study was one of multiple studies that utilized this macaque preparation, not every region was available for sampling from every animal.

### Pregnant human subjects

In this study, the human amnion mRNA data for AQP1, AQP3, AQP8, AQP9, and AQP11 were obtained from subjects with normal non‐diabetic term pregnancies (*n* = 20), as reported in part in our recent study (Bednar et al. 2015). The study design was approved by the Institution Review Board of Oregon Health and Science University (OHSU). Written Informed Consent and HIPPA Research Authorization was obtained from all subjects that participated in the study. Experimental protocols and mRNA quantification methodologies have been described in detail elsewhere (Bednar et al. 2015).

### Real time RT‐qPCR for macaque aquaporins

The AQP mRNA levels in macaque amnions were determined by real‐time RT‐qPCR. Amnion tissues collected immediately at necropsy were placed in RNA*later* ^*®*^ (InVitrogen, Thermo Fisher Scientific) for RNA extraction using an RNeasy Kit (Qiagen, Inc., Valencia, CA).

Total RNA (0.5 *μ*g) was reversed transcribed using MultiScribe reverse transcriptase. Sample cDNA was amplified using custom designed *Macaca mulatta*‐specific primers for AQP1 (Primer Express^®^ Software v3.0. Applied Biosystems, Thermo Fisher Scientific). Pre‐designed *Macaca mulatta*‐specific primers were used for AQP3, AQP8, AQP9, and AQP11 (Applied Biosystems, Thermo Fisher Scientific) (Table 1). PES1 (pescadillo ribosomal biogenesis factor 1) was used as the house‐keeping gene.

**Table 1:** *Macaca mulatta*‐specific aquaporin primers^a^

Gene	Assay ID/sequence	Source
AQP1^b^	A189LG2 RhAQP1_110514 Forward: 5′–CGCGGTGATCACACACAACT‐3′ Reverse: 5′–GGGCTCCCCCGATGAAT‐3′ (68 bp)	Custom designed
AQP3	Rh02856041_m1 AQP3 FAM	Pre‐designed
AQP8	Rh02837748_m1 AQP8 FAM	Pre‐designed
AQP9	Rh02878553_m1 AQP9 FAM	Pre‐designed
AQP11	Rh02887164_m1 AQP11 FAM	Pre‐designed

a AQP primers conjugated to dye label FAM were pre‐designed from Applied Biosystems. Sequence information and amplicon size were proprietary.

b AQP1 primers were custom‐designed (using Primer Express^®^ Software v3.0., Applied Biosystems, Thermo Fisher Scientific).

The relative quantity of amnion mRNA was measured by real‐time qPCR using a PE Applied Biosystems PRISM 7900 Sequences Detector System (InVitrogen Life Technologies, Carlsbad, CA). The amplification was performed as follows: 2 min at 50°C, 10 min at 95°C, followed by 45 cycles each at 95°C for 15 sec, and at 60°C for 60 sec. Standard curves on serial dilutions of the cDNA (1:2, 1:5, 1:20, 1:50, 1:200 1:500, and 1:2000) were performed for each AQP and PES1 using cDNA from pooled macaque amnion samples. Standard curves were drawn on the basis of the log of the input RNA versus the critical threshold (CT) cycle, in which the fluorescence of the sample was greater than the threshold of the baseline fluorescence. Quantitative PCR results were normalized to PES1 housekeeping gene expression. The relative quantities of AQP mRNA were determined using the 2^−∆∆CT^ method (Bednar et al. 2015). To do this, using the mean CT values from triplicate determinations of AQP1, AQP3, AQP8, AQP9, and AQP11 and the internal reference PES1, the ∆CT values were calculated for each AQP by subtracting the associated PES1 CT means. The mean AQP1 ∆CT in amnion of control diet animals was used as the calibrator to obtain the ∆∆CT values for each AQP. Calculation of 2^−∆∆CT^ yields the relative quantity of mRNA normalized to the control diet AQP1 levels in the amnion.

### Statistical analysis

Data are presented as mean ± SE and were statistically analyzed using *t*‐tests as well as one and two factor analyses of variance (1‐ factor and 2‐factor ANOVA). Post hoc testing utilized Fisher's least significant difference for multiple comparisons if the ANOVA null hypothesis was rejected. The interaction term of a 2‐factor ANOVA was used to determine differences among expression profiles of the five AQPs. Relationships between variables were determined by bivariate and multivariate linear regression. For regression of AFI against mRNA level of each AQP, the same AFI value was used for each region of the amnion if multiple regions of the amnion were analyzed. Data were logarithmically transformed prior to statistical analysis to normalize variances as needed. *P* < 0.05 was considered significant.

## Results

In the macaques, maternal weight in the control diet group (11.1 ± 0.69 kg) was not different than that in the HFD group (11.6 ± 1.1 kg).

**Figure 1:**
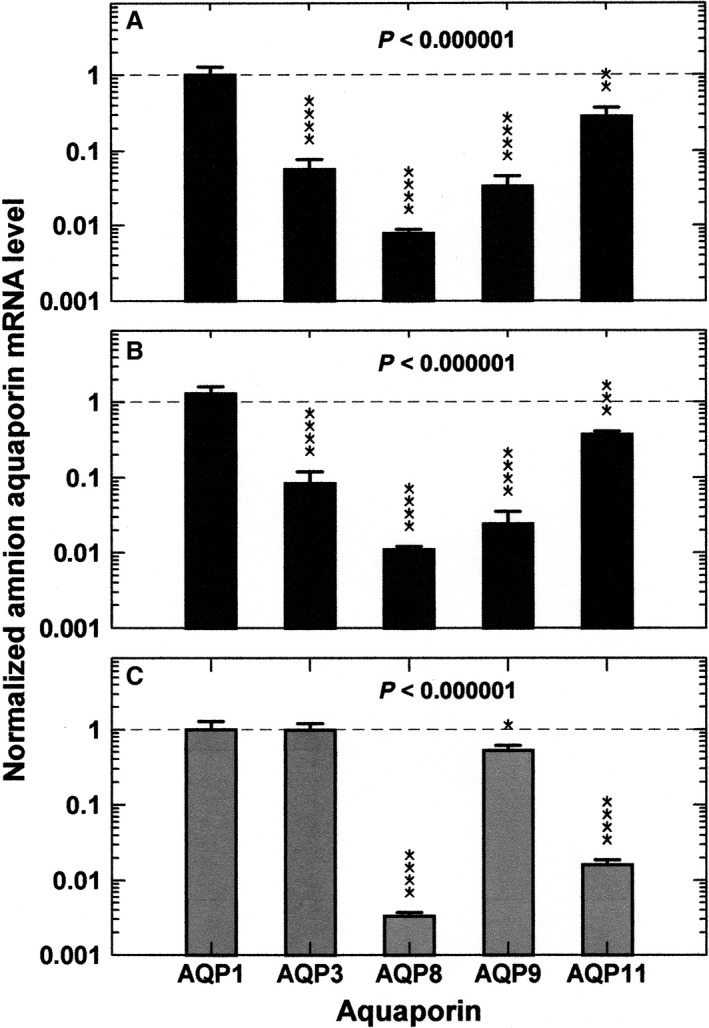
Aquaporin 1, AQP3, AQP8, AQP9, and AQP11 mRNA levels in the amnion of Japanese macaques from all amnion regions combined (mean ± SE) under, (A) control diet (*n* = 8) and (B) HFD (B, *n* = 10) conditions. Individual AQP mRNA values are normalized to mean AQP1 mRNA levels of control diet (dashed line). (C) Aquaporin mRNA levels in human amnion of normal non‐diabetic subjects (*n* = 20) from our recent study (Bednar et al. 2015). The data are normalized to mean human AQP1 mRNA (dashed line) from placental and reflected amnion combined. The *P* values from 1‐factor ANOVA are shown. *P <* *0.05, **P <* *0.01, ***P <* *0.001, and ****P <* *0.0001 for each AQP compared to the respective AQP1 mRNA levels.

Five AQPs were detected in the macaque amnion and their relative mRNA levels are shown in Figure 1. In control diet animals, highly significant differences in amnion mRNA levels were observed among the AQPs where AQP3, AQP8, AQP9, and AQP11 mRNA levels were significantly lower than that of AQP1. The largest difference was found for AQP8 at a level 120 fold lower as compared to AQP1 (Fig. 1A). The mRNA profile of the five AQPs in the amnion of non‐obese HFD animals was similar to that in the control diet animals (Fig. 1B. 2‐factor ANOVA, interaction *P *=* *0.79 comparing the two groups). By individual AQP comparisons, similarly there were no statistically significant differences in mRNA levels between the control and HFD groups. When these values in the macaque amnion were compared with AQP values in the amnion of normal non‐diabetic human subjects (Fig. 1C), the profile of the five AQPs was significantly different for both the control diet (2‐factor ANOVA, interaction *P *<* *0.001, control macaque vs. human) and the HFD (2‐factor ANOVA, interaction *P *<* *0.001, HFD macaque vs. human) animals. By post hoc testing, macaque mRNA levels for AQP3 and AQP9 were significantly lower (*P *<* *0.05) while AQP8 and AQP11 mRNA significantly higher (*P *<* *0.01) than human levels relative to the respective mean AQP1 values.

**Figure 2:**
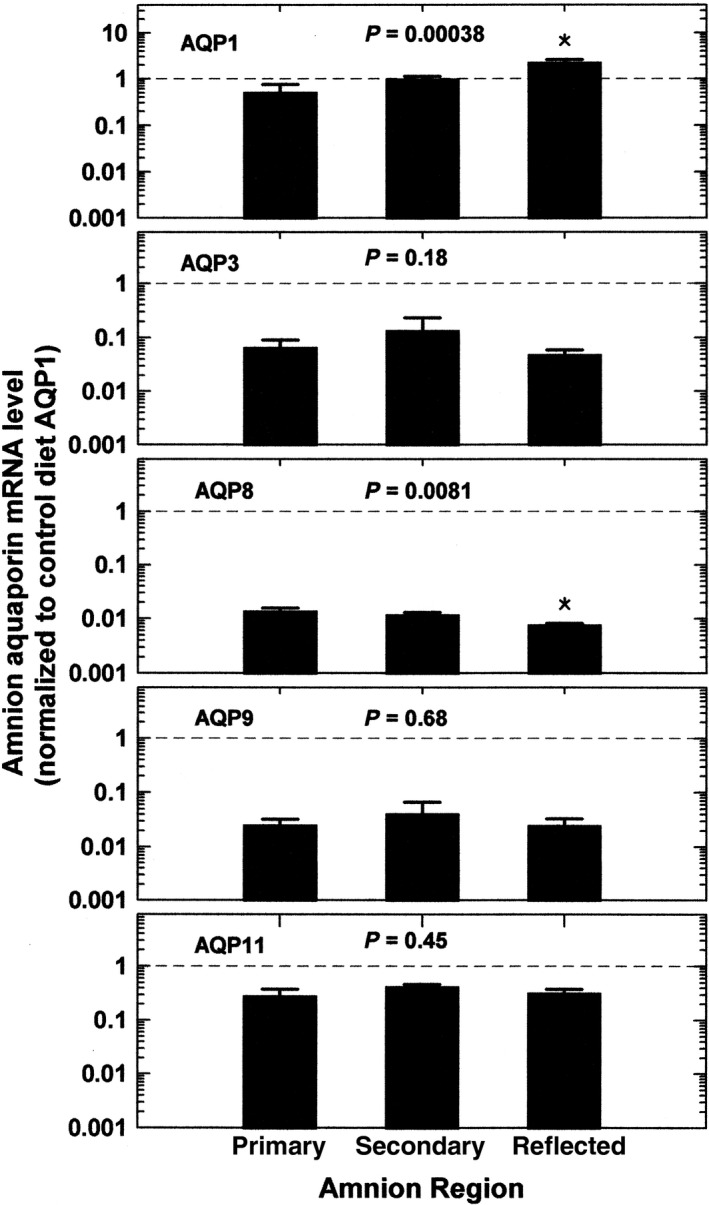
Regional differences of AQP expression profiles in amnion of control and HFD macaques. Data shown for each AQP mRNA are combined control and HFD groups normalized to the combined mean AQP1 level for the primary placental, secondary placental, and reflected amnion (dashed lines). One factor ANOVA *P* values are shown. *P <* *0.05 compared to the respective AQP in primary placental amnion.

**Figure 3:**
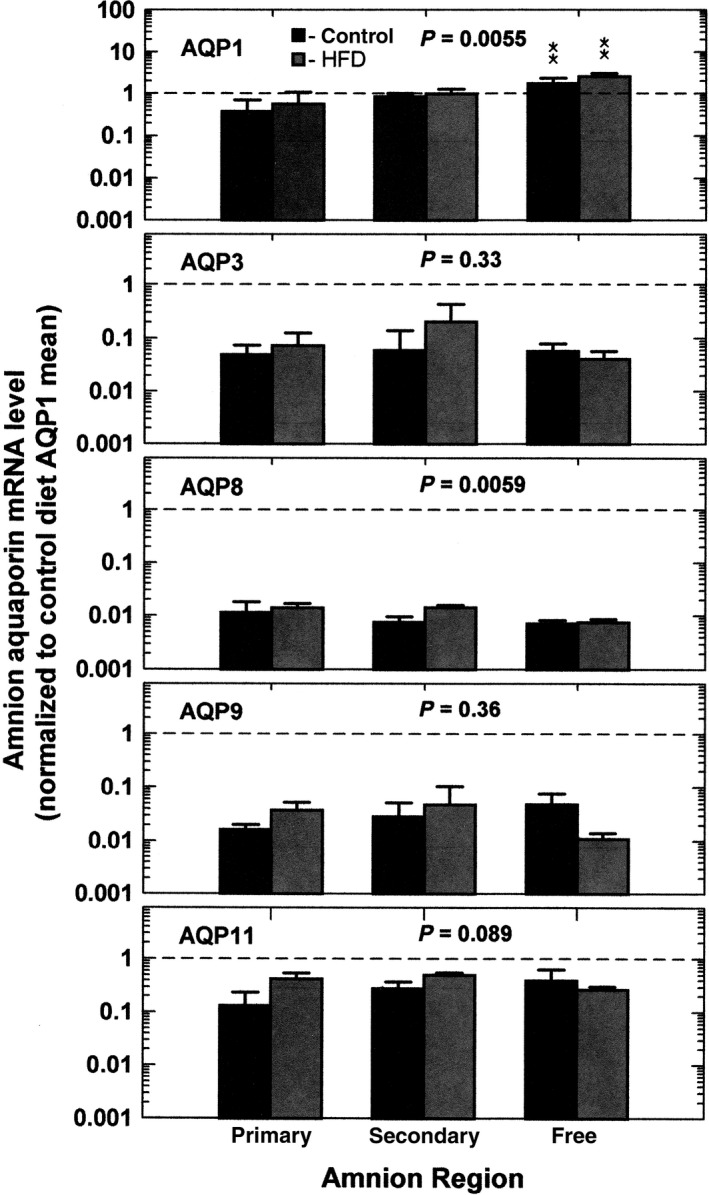
Regional variations in AQP mRNA expression subdivided into control and HFD in the primary placental, secondary placental, and reflected amnion. Data for individual AQPs are normalized to mean AQP1 mRNA level (dashed line) from all regions and diets combined. The interaction *P* values are from 2‐factor ANOVA comparing effects of diet. **P <* *0.01 compared to respective AQP mRNA levels in primary amnion of control diet.

When the control and HFD groups were combined, there were regional differences in AQP1 (1‐factor ANOVA, *P *=* *0.00038) and AQP8 (*P *=* *0.0081) mRNA levels (Fig. 2). AQP1 mRNA level was highest in the reflected amnion (*P *<* *0.05) while AQP8 was lowest in the reflected amnion (*P *<* *0.05) as compared to that in the primary placental amnion. AQP3, AQP9, and AQP11 mRNA levels did not vary among amnion regions. When subdivided according to diet, HFD significantly increased AQP1 (2‐factor ANOVA, *P *=* *0.0055) and AQP8 (*P *=* *0.0059) mRNA levels in all three amnion regions (Fig. 3). By post hoc testing, the regional differences for AQP1 (*P *<* *0.01) persisted with levels highest in the reflected amnion for both control and HFD groups. For AQP8, regional differences in the two groups were not significant with post hoc testing (Fig. 3).

**Figure 4:**
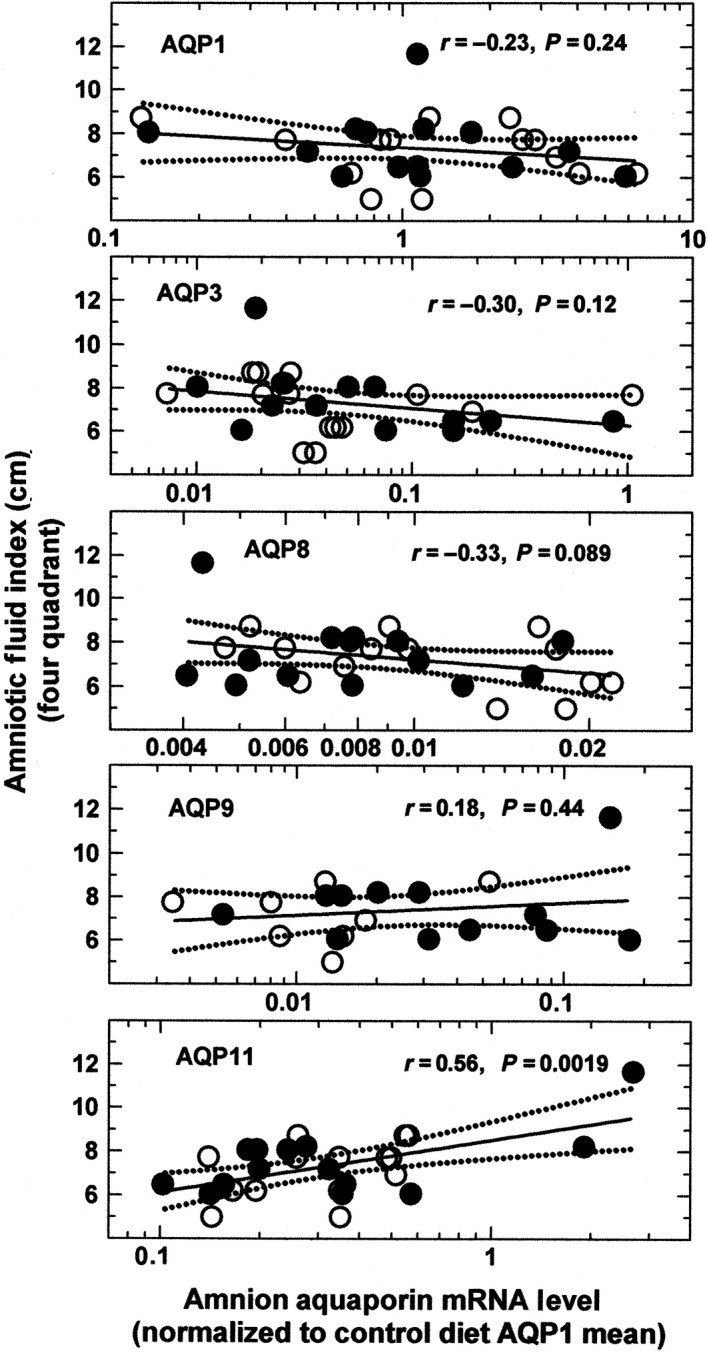
Regression relationships between the amniotic fluid index (AFI) and mRNA level of each AQP in amnion (all regions combined) of control (filled circles) and HFD (open circles) macaques. Solid lines are the regression lines and dotted lines are the 95% confidence interval about the regression line. Correlation coefficient and *P* values are shown for individual AQPs. Note that scales on the horizontal axis are unique for each AQP.

The mean AFI in the HFD animals was slightly lower than that in the control diet group however, the AFI in the control (7.54 ± 0.66 cm, *n* = 8) and HFD (7.16 ± 0.46 cm, *n* = 7) animals were not statistically different (*P *=* *0.66). The AFI was not correlated with maternal weight independent of maternal diet status. When regressed against AQP mRNA levels using bivariate regression, the AFI was positively correlated with AQP11 (*P *=* *0.0019) but not with the remaining AQPs (Fig. 4). Using multivariate regression, AFI was again correlated only with AQP11 mRNA levels.

## Discussion

Although speculated to be important regulators of AFV since their initial discovery in fetal membranes (Liu and Wintour 2005), the physiologic significance of the AQPs in the maintenance and regulation of amniotic fluid transport has yet to be established. Furthermore, it is not known whether species variations in AQP expressions in the amnion would lead to differences in functional responses to physiological or pathological alterations in AFV. In this study, we demonstrated that in Japanese macaques, five AQPs were expressed in all regions of the amnion with mRNA levels of AQP1 and AQP3 being highest and AQP8 lowest among the five AQPs. This observation is comparable to that in human amnion shown in our recent study (Bednar et al. 2015). However, the expression profiles of the five AQPs in macaque amnion of both control and HFD animals differed from that in human amnion of normal non‐diabetic subjects. Our analysis showed that AQP3 and AQP9 mRNA levels were significantly lower and AQP8 and AQP11 mRNA levels higher in macaque than in human. In addition, in human amnion, there were regional differences in expression of all five AQPs in that levels were lower in the reflected compared to placental amnion (Bednar et al. 2015). In the macaque there were minimal regional differences for AQP3, AQP9, and AQP11 among the primary placental, secondary placental, and reflected amnions while AQP1 was highest and AQP8 lowest in the reflected placental amnion. These findings are significant in that, even among primates, there are species‐specific differences in AQP gene expressions in the amnion. It is noteworthy that the sheep amnion similarly expresses these five AQPs although the expression profile is significantly different from that in the macaque and human amnion. In ovine amnion, AQP8 mRNA level is highest while AQP9 is lowest when compared to AQP1, AQP3 and AQP11 levels (Cheung et al. 2016). Whether such differences would alter amniotic fluid dynamics is unknown.

In this study, the HFD appeared to be associated with increased mRNA levels of AQP1 and AQP8 in the macaque amnion. This suggests that amnion water transport could potentially be affected since AQP1 is a high efficiency water channel (Agre et al. 1993), and to a lesser extent AQP8 (Koyama et al. 1998; Soria et al. 2010), in facilitating water transfer. However, since there was no correlation between AFI and amnion AQP1 or AQP8 mRNA levels, AQPs may not be the primary determinant of AFV in the macaques. The positive correlation between AFI and AQP11 is of interest, although a water transport function for AQP11 has not been clearly established since AQP11 is primarily expressed in the testis with a function yet to be defined (Gorelick et al. 2006; Yakata et al. 2011). Whether AQP11 would significantly alter AFV by modifying amniotic fluid transport would require further investigation.

The aquaglyceroporins AQP3 and AQP9 are transmembrane proteins that transport glycerol in addition to water. Aquaporin 3 is involved in glycerol transport in adipose tissues (Rodríguez et al. 2011) and AQP9 is a specific glycerol transport channel in the liver. Hepatic AQP9 facilitates uptake of glycerol for synthesis of glucose and triglycerides thus involved in the pathogenesis of obesity and diabetes. Gene expression of AQP9 in the liver is up‐regulated in streptozotocin‐induced diabetic rats (Carbrey et al. 2003) and mice (Kuriyama et al. 2002), as well as in diet‐induced obese mice (Hirako et al. 2016). In the present study, amnion AQP3 and AQP9 mRNA levels in HFD resistant macaques were not different from that in control animals. The lack of effect of high fat intake on AQP3 and AQP 9 gene expression most likely is due to the resistant phenotype in which the animals remain lean with normal insulin function. Therefore, in the absence of obesity and diabetes, dietary high fat content alone does not appear to have significant effects on gene expression of the aquaglyceroporins. Additionally, the amnion is not known to be a major tissue important for lipogenesis.

Our present study examined the relationships between macaque amnion AQP mRNA levels and maternal dietary status. We further analyzed the correlation of AQP gene expression and AFV in control and HFD groups. However, gene expression levels may not reflect biological functions whereas proteins are the effectors that mediate functions. The translation of genes from mRNA into functional proteins is a complex process involving multiple levels of translational regulation as well as post‐translational modifications (Arcondéguy et al. 2013). The absence of protein data is a potential pitfall in this study. However, since this study was one of multiple studies that utilized this macaque preparation, as such, the amount of amnion tissues available for the present study was limited and insufficient for protein analysis in addition to mRNA quantification. In our previous study of AQP gene expressions in human amnion (Bednar et al. 2015), we found a modest positive correlation (*r* ^2^ × 100% = 32%) between AQP mRNA and protein levels. Thus, the present gene expression data in macaques could provide valuable insights into the role of AQPs in AFV regulation under conditions of high fat intake.

In view of the increasing prevalence of HFD and obesity in the general population, it is important to consider whether a diet high in fat could adversely affect AFV in pregnancy. In sheep, maternal obesity reduced both amniotic and allantoic fluid volumes as compared to control non‐obese animals (Satterfield et al. 2012). In our previous study in pregnant women (Bednar et al. 2015), the AFI tended to decrease as BMI increased in control non‐diabetic subjects although the dietary status of these subjects was not known. In this study, a HFD in pregnant macaques that did not develop obesity was not associated with significant decreases in the AFI and there was no correlation between maternal weight and AFI. Although our results appear to indicate changes in amnion AQP1 and AQP8 expression under HFD conditions, the resulting changes in amniotic water transport may not have been reflected as changes of AFI. Collectively, this study suggests that a diet high in fat without development of obesity may have relatively minor effects on AFV and AQP expressions in the amnion. Thus, a maternal high fat resistant phenotype in the absence of obesity and diabetes may be protective against certain adverse effects of HFD during pregnancy.

## Conflict of Interest

The authors declare that there are no conflicts of interest, and there are no disclosures to declare.

## References

[phy213792-bib-0001] Abele, H. , S. Starz , M. Hoopmann , B. Yazdi , K. Rall , and K. O. Kagan . 2012 Idiopathic polyhydramnios and postnatal abnormalities. Fetal Diagn. Ther. 32:251–255. 22760013 10.1159/000338659

[phy213792-bib-0002] Agre, P. , G. M. Preston , B. L. Smith , J. A. Jung , S. Raina , C. Moon , et al. 1993 Aquaporin CHIP: the archetypal molecular water channel. Am. J. Physiol. Renal Physiol. 265:F463–F476. 10.1152/ajprenal.1993.265.4.F463 7694481

[phy213792-bib-0003] Arcondéguy, T. , E. Lacazette , S. Millevoi , H. Prats , and C. Touriol . 2013 VEGF‐A mRNA processing, stability and translation: a paradigm for intricate regulation of gene expression at the post‐transcriptional level. Nucleic Acids Res. 41:7997–8010. 23851566 10.1093/nar/gkt539 PMC3783158

[phy213792-bib-0004] Beall, M. H. , J. P. van den Wijngaard , M. J. van Gemert , and M. G. Ross . 2007 Amniotic fluid water dynamics. Placenta 28:816–823. 17254633 10.1016/j.placenta.2006.11.009

[phy213792-bib-0005] Bednar, A. D. , M. K. Beardall , R. A. Brace , and C. Y. Cheung . 2015 Differential expression and regional distribution of aquaporins in amnion of normal and gestational diabetic pregnancies. Physiol. Rep. 3:e12320 10.14814/phy2.12320. 25742957 PMC4393155

[phy213792-bib-0006] Brace, R. A. , and C. Y. Cheung . 2014 Regulation of amniotic fluid volume: evolving concepts. Adv. Exp. Med. Biol. 814:49–68. 25015800 10.1007/978-1-4939-1031-1_5

[phy213792-bib-0008] Brace, R. A. , D. F. Anderson , and C. Y. Cheung . 2014 Regulation of amniotic fluid volume: mathematical model based on intramembranous transport mechanisms. Am. J. Physiol. Regul. Integr. Comp. Physiol. 307:R1260–R1273. 25186112 10.1152/ajpregu.00283.2014 PMC4233290

[phy213792-bib-0009] Carbrey, J. M. , D. A. Gorelick‐Feldman , D. Kozono , J. Praetorius , S. Nielsen , and P. Agre . 2003 Aquaglyceroporin AQP9: solute permeation and metabolic control of expression in liver. Proc. Natl. Acad. Sci. USA 100:2945–2950. 12594337 10.1073/pnas.0437994100 PMC151446

[phy213792-bib-0010] Cheung, C. Y. , D. F. Anderson , and R. A. Brace . 2016 Aquaporins in ovine amnion: responses to altered amniotic fluid volumes and intramembranous absorption rates. Physiol. Rep. 4:e12868 10.14814/phy2.12868. 27440743 PMC4962073

[phy213792-bib-0011] Contu, L. , and C. A. Hawkes . 2017 A review of the impact of maternal obesity on the cognitive function and mental health of the offspring. Int. J. Mol. Sci. 18:e1093 10.3390/ijms18051093. 28534818 PMC5455002

[phy213792-bib-0012] Frias, A. E. , and K. L. Grove . 2012 Obesity: a transgenerational problem linked to nutrition during pregnancy. Semin. Reprod. Med. 30:472–478. 23074005 10.1055/s-0032-1328875 PMC3615704

[phy213792-bib-0013] Frias, A. E. , T. K. Morgan , A. E. Evans , J. Rasanen , K. Y. Oh , K. L. Thornburg , et al. 2011 Maternal high‐fat diet disturbs uteroplacental hemodynamics and increases the frequency of stillbirth in a nonhuman primate model of excess nutrition. Endocrinology 152:2456–2464. 21447636 10.1210/en.2010-1332 PMC3100625

[phy213792-bib-0014] Gilbert, W. M. , E. Eby‐Wilkens , and A. F. Tarantal . 1997 The missing link in rhesus monkey amniotic fluid volume regulation: intramembranous absorption. Obstet. Gynecol. 89:462–465. 9052606 10.1016/S0029-7844(96)00509-1

[phy213792-bib-0015] Gorelick, D. A. , J. Praetorius , T. Tsunenari , S. Nielsen , and P. Agre . 2006 Aquaporin‐11: a channel protein lacking apparent transport function expressed in brain. BMC Biochem. 7:14 10.1186/1471-2091-7-14. 16650285 PMC1475587

[phy213792-bib-0016] Grayson, B. E. , P. R. Levasseur , S. M. Williams , M. S. Smith , D. L. Marks , and K. L. Grove . 2010 Changes in melanocortin expression and inflammatory pathways in fetal offspring of nonhuman primates fed a high‐fat diet. Endocrinology 151:1622–1632. 20176722 10.1210/en.2009-1019 PMC2850229

[phy213792-bib-0017] Grigsby, P. L. 2016 Animal models to study placental development and function throughout normal and dysfunctional human pregnancy. Semin. Reprod. Med. 34:11–16. 26752715 10.1055/s-0035-1570031 PMC4799492

[phy213792-bib-0018] Hamza, A. , D. Herr , E. F. Solomayer , and G. Meyberg‐Solomayer . 2013 Polyhydramnios: causes, diagnosis and therapy. Geburtshilfe Frauenheilkd. 73:1241–1246. 24771905 10.1055/s-0033-1360163 PMC3964358

[phy213792-bib-0019] Harris, R. A. , C. E. Alcott , E. L. Sullivan , D. Takahashi , C. E. McCurdy , S. Comstock , et al. 2016 Genomic variants associated with resistance to high fat diet induced obesity in a primate model. Sci. Rep. 6:36123 10.1038/srep36123. 27811965 PMC5095882

[phy213792-bib-0020] Hirako, S. , Y. Wakayama , H. Kim , Y. Iizuka , A. Matsumoto , N. Wada , et al. 2016 The relationship between aquaglyceroporin expression and development of fatty liver in diet‐induced obesity and ob/ob mice. Obes. Res. Clin. Pract. 10:710–718. 26747210 10.1016/j.orcp.2015.12.001

[phy213792-bib-0021] Koyama, N. , K. Ishibashi , M. Kuwahara , N. Inase , M. Ichioka , S. Sasaki , et al. 1998 Cloning and functional expression of human aquaporin8 cDNA and analysis of its gene. Genomics 54:169–172. 9806845 10.1006/geno.1998.5552

[phy213792-bib-0022] Kuriyama, H. , I. Shimomura , K. Kishida , H. Kondo , N. Furuyama , H. Nishizawa , et al. 2002 Coordinated regulation of fat‐specific and liver‐specific glycerol channels, aquaporin adipose and aquaporin 9. Diabetes 51:2915–2921. 12351427 10.2337/diabetes.51.10.2915

[phy213792-bib-0023] Liu, H. , and E. M. Wintour . 2005 Aquaporins in development – a review. Reprod. Biol. Endocrinol. 3:18 10.1186/1477-7827-3-18. 15888206 PMC1156947

[phy213792-bib-0024] Locatelli, A. , P. Vergani , L. Toso , M. Verderio , J. C. Pezzullo , and A. Ghidini . 2004 Perinatal outcome associated with oligohydramnios in uncomplicated term pregnancies. Arch. Gynecol. Obstet. 269:130–133. 12928935 10.1007/s00404-003-0525-6

[phy213792-bib-0025] Lowensohn, R. I. , D. D. Stadler , and C. Naze . 2016 Current concepts of maternal nutrition. Obstet. Gynecol. Surv. 71:413–426. 27436176 10.1097/OGX.0000000000000329 PMC4949006

[phy213792-bib-0026] Mann, S. E. , E. A. Ricke , E. A. Torres , and R. N. Taylor . 2005 A novel model of polyhydramnios: amniotic fluid volume is increased in aquaporin 1 knockout mice. Am. J. Obstet. Gynecol. 192:2041–2044. 15970890 10.1016/j.ajog.2005.02.046

[phy213792-bib-0027] McCurdy, C. E. , J. M. Bishop , S. M. Williams , B. E. Grayson , M. S. Smith , J. E. Friedman , et al. 2009 Maternal high‐fat diet triggers lipotoxicity in the fetal livers of nonhuman primates. J. Clin. Invest. 119:323–335. 19147984 10.1172/JCI32661 PMC2631287

[phy213792-bib-0028] McCurdy, C. E. , S. Schenk , B. Hetrick , J. Houck , B. G. Drew , S. Kaye , et al. 2016 Maternal obesity reduces oxidative capacity in fetal skeletal muscle of Japanese macaques. J. C. I. Insight 1:e86612 10.1172/jci.insight.86612. PMC5053156 27734025

[phy213792-bib-0029] McMahon, M. J. , C. V. Ananth , and R. M. Liston . 1998 Gestational diabetes mellitus. Risk factors, obstetric complications and infant outcomes. J. Reprod. Med. 43:372–378. 9583071

[phy213792-bib-0030] Morris, R. K. , C. H. Meller , J. Tamblyn , G. M. Malin , R. D. Riley , M. D. Kilby , et al. 2014 Association and prediction of amniotic fluid measurements for adverse pregnancy outcome: systematic review and meta‐analysis. BJOG 121:686–699. 24738894 10.1111/1471-0528.12589

[phy213792-bib-0031] Nicol, L. E. , W. F. Grant , S. M. Comstock , M. L. Nguyen , M. S. Smith , K. L. Grove , et al. 2013 Pancreatic inflammation and increased islet macrophages in insulin‐resistant juvenile primates. J. Endocrinol. 217:207–213. 23420316 10.1530/JOE-12-0424 PMC3697080

[phy213792-bib-0032] O'Tierney‐Ginn, P. , V. Roberts , M. Gillingham , J. Walker , P. A. Glazebrook , K. L. Thornburg , et al. 2015 Influence of high fat diet and resveratrol supplementation on placental fatty acid uptake in the Japanese macaque. Placenta 36:903–910. 26145226 10.1016/j.placenta.2015.06.002 PMC4529757

[phy213792-bib-0033] Pound, L. D. , S. M. Comstock , and K. L. Grove . 2014 Consumption of a Western‐style diet during pregnancy impairs offspring islet vascularization in a Japanese macaque model. Am. J. Physiol. Endocrinol. Metab. 307:E115–E123. 24844258 10.1152/ajpendo.00131.2014 PMC4080145

[phy213792-bib-0034] Prat, C. , L. Blanchon , V. Borel , D. Gallot , A. Herbet , D. Bouvier , et al. 2012 Ontogeny of aquaporins in human fetal membranes. Biol. Reprod. 86(48):1–8. 10.1095/biolreprod.111.095448 22053096

[phy213792-bib-0035] Roberts, V. H. , A. E. Frias , and K. L. Grove . 2015 Impact of maternal obesity on fetal programming of cardiovascular disease. Physiology (Bethesda) 30:224–231. 25933822 10.1152/physiol.00021.2014 PMC4422977

[phy213792-bib-0036] Rodríguez, A. , V. Catalán , J. Gómez‐Ambrosi , S. García‐Navarro , F. Rotellar , V. Valentí , et al. 2011 Insulin‐ and leptin‐mediated control of aquaglyceroporins in human adipocytes and hepatocytes is mediated via the PI3K/Akt/mTOR signaling cascade. J. Clin. Endocrinol. Metab. 96:E586–E597. 21289260 10.1210/jc.2010-1408

[phy213792-bib-0037] Satterfield, M. C. , K. A. Dunlap , D. H. Keisler , F. W. Bazer , and G. Wu . 2012 Arginine nutrition and fetal brown adipose tissue development in diet‐induced obese sheep. Amino Acids 43:1593–1603. 22327565 10.1007/s00726-012-1235-9

[phy213792-bib-0038] Soria, L. R. , E. Fanelli , N. Altamura , M. Svelto , R. A. Marinelli , and G. Calamita . 2010 Aquaporin‐8‐facilitated mitochondrial ammonia transport. Biochem. Biophys. Res. Commun. 393:217–221. 20132793 10.1016/j.bbrc.2010.01.104

[phy213792-bib-0039] Yakata, K. , K. Tani , and Y. Fujiyoshi . 2011 Water permeability and characterization of aquaporin‐11. J. Struct. Biol. 174:315–320. 21251984 10.1016/j.jsb.2011.01.003

